# Cost-effectiveness of companion BRCA testing and adjuvant olaparib treatment in patients with BRCA-mutated high-risk HER2-negative early breast cancer

**DOI:** 10.3389/fonc.2025.1419071

**Published:** 2025-04-15

**Authors:** Silviya Nikolova, Wenkang Ma, Gurdeep S. Sagoo, Yogesh Punekar, Elizabeth Sheppard

**Affiliations:** ^1^ Health Economics and Outcomes Research (HEOR) Ltd, Cardiff, United Kingdom; ^2^ Lumanity, London, United Kingdom; ^3^ Population Health Sciences Institute, Faculty of Medical Sciences, Newcastle University, Newcastle, United Kingdom; ^4^ IQVIA, EMEA Real World Methods & Evidence Generation, London, United Kingdom; ^5^ Oncology Diagnostics, AstraZeneca, Cambridge, United Kingdom

**Keywords:** precision medicine, companion diagnostics, cost effectiveness analysis, targeted therapy, biomarker testing

## Abstract

**Background and objective:**

As the healthcare industry evolves towards precision medicine, methods to assess the value of integrating companion diagnostics in clinical practice through adequate reimbursement levels are becoming essential. Cost-effectiveness analysis is an established tool used to inform the reimbursement of health technologies.

**Methods:**

A decision-tree model was developed to estimate the incremental cost-effectiveness of companion BRCA testing and olaparib use versus no testing and the standard of care (SoC) for patients with BRCA-mutated high-risk HER2-negative early breast cancer from a UK NHS/PSS perspective.

**Results:**

BRCA testing combined with treatment with adjuvant olaparib was associated with an incremental cost-effectiveness ratio (ICER) of £49,327 per quality-adjusted life-year (QALY) gained and an ICER of £86,349 per QALY gained for patients with triple-negative breast cancer (TNBC) and human epidermal growth factor receptor 2 negative/hormone receptor-positive (HER2-/HR+) breast cancer, respectively, compared to no testing and treatment with SoC. This difference in ICER is due to significantly improved outcomes for patients with TNBC who were treated with targeted therapy. For both patient subgroups with early breast cancer, testing and olaparib improved patient outcomes and, despite its relatively high cost, the test and treat strategy was deemed to represent an acceptable use of resources.

**Conclusions:**

The advancement of high-throughput sequencing technologies, coupled with the rise of targeted treatments in recent years, has facilitated a shift from conventional medical practices to individualized oncology therapeutic approaches. Our analysis presented the value of combining genetic sequencing and targeted therapy for patients with breast cancer carrying BRCA mutations and also provided the prototype of a testing model that can be utilized to promote precision medicine for better patient outcomes.

## Introduction

Current access to high-quality oncology biomarker testing is inconsistent across European countries. A key barrier that limits patient access to biomarker testing in Europe is the desynchronized regulatory approval and reimbursement of the cost of the medicines and the companion diagnostics (CDx). *In vitro* diagnostics and pharmaceutical drugs traditionally follow separate routes to patient access at the level of health technology agencies. Targeted pharmaceutical drugs, however, conditional on biomarker status, can only be used after biomarker testing, which inevitably links a pharmaceutical drug to a CDx. Testing is only ordered in cases if it can support a corresponding treatment decision, and the precision medicine (PM) is reimbursed but the associated CDx is not.

The diagnostic regulatory landscapes in four countries in the European Union (EU) (Germany, France, Italy, and Spain) and the United Kingdom (UK) are markedly different and, thus, obtaining reimbursement for testing calls for a country-specific approach. The European Medicines Agency (EMA) in the EU and the Medicines and Healthcare products Regulatory Agency (MHRA) in the UK both require conformity assessment routes to be followed for new genetic testing methods to enter the market (1, 2). The National Institute for Health and Care Excellence (NICE) developed the Diagnostics Assessment Program (DAP), a specific diagnostics healthcare technology assessment (HTA) process. The framework provides methodological guidance for stakeholders and decision-makers. However, it is not linked to reimbursement decisions (3). In Germany, CDx do not undergo HTA, though reimbursement requirements and structures are well defined. CDx reimbursement is provided at a national level for patients with statutory health insurance, but reimbursement pathways differ for tests used in outpatient settings [via the doctor’s fee scale, *Einheitlicher Bewertungsmaßstab* (EBM)] versus the inpatient setting [via the diagnosis-related group (DRG) codes]. In France, treatment and CDx are assessed by two different bodies, namely the Transparency Committee (CT) and the Medical Device and Health Technology Evaluation Committee (CNEDiMTS). As an innovative coverage pathway, CDx can be conditionally and temporarily reimbursed ahead of the HTA under the coverage of the evidence development program and registered with the nomenclature for innovative tests (RIHN) which provides temporary reimbursement in exchange for evidence generation (4–6). In Italy, CDx are reimbursed through DRG tariffs in the inpatient (*delle prestazioni ambulatoriali*, NTPO) and outpatient (*delle prestazioni ospedaliere*, NTPA) settings (7). Although the Italian National Agency for Regional Healthcare Services (AGENAS) may perform HTA for diagnostics at a national level, this is not linked to reimbursement, and decisions are made at regional and local levels. There is no explicit pathway for CDx in Spain currently, and vendors notify the Minister of Health (MoH) of the marketing of CDx and negotiate at regional and hospital levels.

As the pharmaceutical industry evolves towards precision medicine, innovative methods to assess the value of integrating CDx into clinical practice through adequate reimbursement levels are becoming essential. Cost-effectiveness analysis is an established method for decision-making to inform the reimbursement of pharmaceuticals and has been used for CDx as well. A few studies have been published for CDx (8–10), although several limitations for these analyses have been noted (11, 12): (a) Many evaluations focused on a pre-selected patient group rather than including all patients regardless of their biomarker status, with results failing to reflect the real clinical setting. (b) Companion biomarker characteristics captured in evaluations were limited to the cost or the accuracy of the test; often, only the costs of testing were modeled, and clinical outcomes and health state utilities were not included due to limited data generated by clinical trials. (c) Poor selection of comparators [e.g., deviating from clinical practice by not using the standard of care (SoC)], while it is well established that cost-effectiveness results depend on the choice of comparators. (d) Heterogeneity between studies for the same test. Although patients’ response to treatment depends on the presence of a particular biomarker, clinical outcome and cost-effectiveness of testing will differ because prognosis differs across tumor sites and even receptor types (TNBC and HR+/HER2- for breast cancer), health gains depend on prognosis, and the total testing costs of diagnosed patients depend on biomarker prevalence within tumor type. These features require that patient outcomes conditional on biomarker presence be used for analysis, and subgroup analysis be considered. (e) The independence of tests used in sequence was often assumed; while the model structure usually reflected the care pathway and progression of the disease, the complexity and constraints of performing a decision analysis on the costs and health outcomes of diagnostic tests make robust modeling challenging, and quality issues remain.

Olaparib, a poly-adenosine diphosphate ribose polymerase (PARP) inhibitor, has been approved by the United States Food and Drug Administration (US FDA) for the treatment of advanced ovarian cancer in patients with germline *BRCA1/2* mutations (gBRCAm) and for the treatment of ERBB2 [previously human epidermal growth factor receptor 2 (HER-2)]-negative metastatic breast cancer associated with a gBRCAm in patients who previously received chemotherapy (13). In the randomized, double-blind OlympiA clinical trial, patients with pathogenic or likely pathogenic gBRCAm and non-metastatic, ERBB2-negative primary breast cancer were randomized to receive twice-daily oral olaparib or placebo for 1 year following completion of definitive local treatment and neoadjuvant or adjuvant chemotherapy (14). In March 2022, an interim analysis with 1,836 patients, 330 invasive-disease-free survival events, and a median of 3.5 years of follow-up reported that patients receiving olaparib had superior 4-year distant disease-free survival (DDFS) (86.5% vs 79.1% for placebo, diff. 7.4%; 95% CI 3.6%, 11.3%) and overall survival (OS) (89.8% vs 86.4% for placebo, diff. 3.4%; 95% CI -0.1%, 6.8%) (15). Olaparib significantly improved OS vs placebo with a hazard ratio (HR) of 0.68 (95% CI 0.47, 0.97; P = 0.009). The HR for distant disease or death at 4 years was 0.61 (95% CI, 0.48, 0.77). Olaparib was subsequently approved for use in the adjuvant setting among patients similar to those in the OlympiA trial (16). A cost-effectiveness analysis reported that adjuvant olaparib was associated with a 1.25-year increase in life expectancy and a 1.20-quality-adjusted life-year (QALY) increase at an incremental cost of $133,133 compared with no olaparib. This corresponded to an incremental cost-effectiveness ratio (ICER) of approximately $111,000 per QALY gained from a US healthcare system perspective. At a willingness-to-pay threshold of $150,000 per QALY, olaparib was cost-effective at its 2021 price and in more than 92% of simulations in a probabilistic sensitivity analysis (17). Adjuvant olaparib is both clinically effective and cost-effective for the treatment of patients with early breast cancer (eBC) with gBRCAm. In this study, we evaluated the value of combining BRCA testing and olaparib treatment for these patients from a UK National Health Service (NHS) and personal social services (PSS) perspective over a patient lifetime horizon using a decision analytical model.

## Methods

### Model

**Figure 1:**
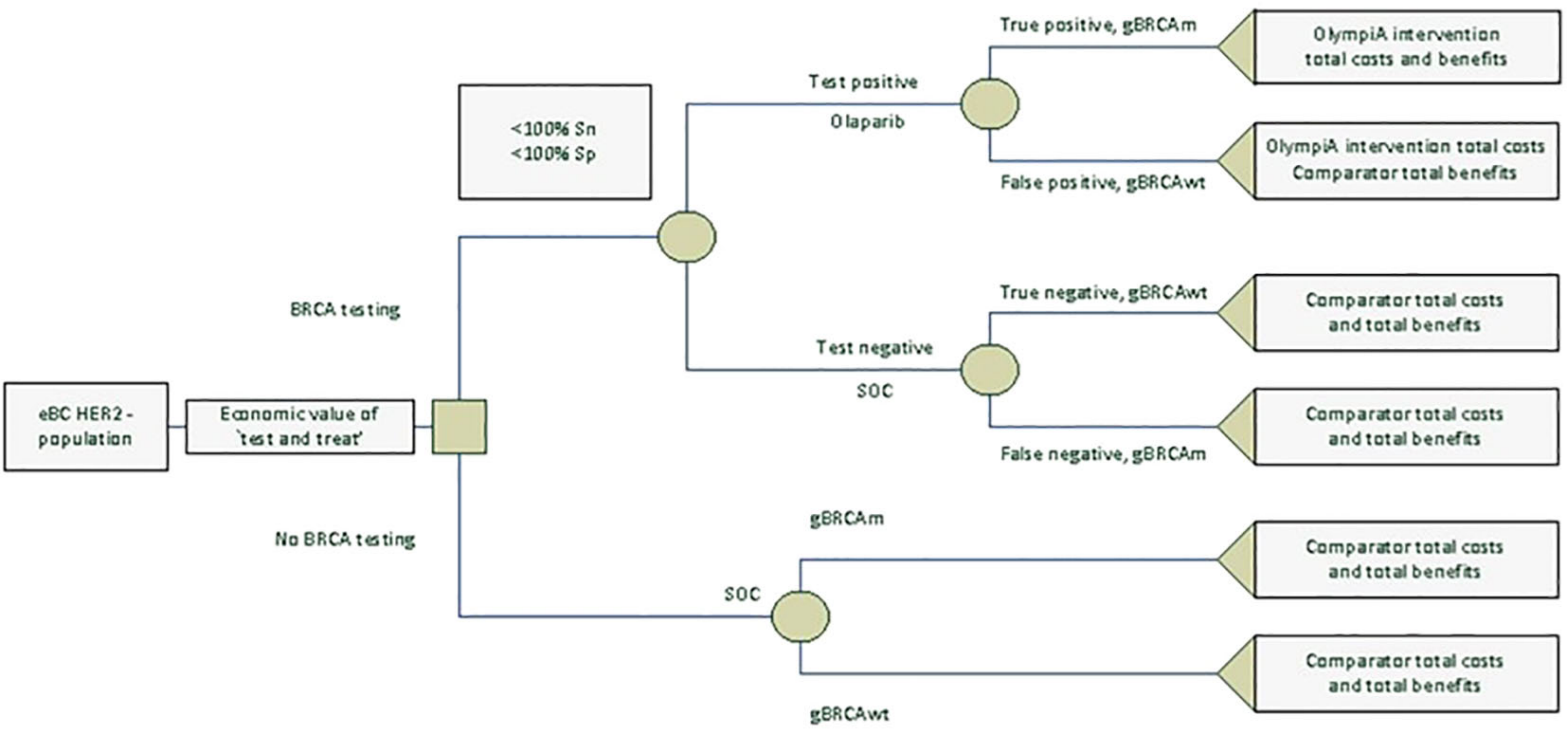
Decision tree testing model.

We created a decision-tree model in Microsoft Excel^®^ to estimate the lifetime costs, life-years (LYs), QALYs, and value for money associated with novel BRCA testing and adjuvant olaparib or no testing and the SoC (or “watch and wait,” proxied by placebo in the OlympiA trial) for patients with eBC that had completed definitive local treatment that includes neoadjuvant or adjuvant chemotherapy from an NHS/PSS perspective. All patients regardless of their gBRCAm status were considered for either the “test and treat” or “no testing” strategy. Among patients who received “test and treat,” olaparib was given to those who tested positive and the SoC to those who tested negative. **Figure 1** shows the model structure. Inputs on the prevalence of gBRCAm, test sensitivity and specificity, and outcomes of patients depending on their gBRCAm status and treatments received are derived from public sources and a cost-effectiveness analysis of olaparib (that used a discounting rate of 3.5% for both cost and health outcomes). Note that due to a lack of clinical and economic evidence for olaparib for patients with gBRCA-wildtype (gBRCAwt) eBC, costs and health benefits for the intent-to-treat (ITT) population from the cost-effectiveness analysis were used for “false positive” and “true negative” patients. We followed the Consolidated Health Economic Evaluation Reporting Standards (CHEERS) reporting guidelines (18). For implementation in the UK NHS, when interpreting the outcome of economic evaluations, NICE generally considers new drugs or health technologies good value for money, and thus suitable for funding within the NHS, if they cost in the range of £20,000 to £30,000 per additional QALY gained (19).

### Inputs

Two populations were analyzed, triple-negative breast cancer (TNBC) and HER2-/hormone receptor-positive (HR+) breast cancer. **Table 1** presents the base case inputs and the distributions used for the probabilistic analysis. Patients who were tested formed four mutually exclusive groups, the share of which was calculated using gBRCAm prevalence and BRCA test specificity and sensitivity:

**Table 1:** Model inputs.

Variable	Patients with TNBC	Patients with HER2-/HR+BC	Distribution for probabilistic analysis	Reference
Epidemiology and test inputs				
Test sensitivity	99%	99%	Beta	Diaceutics (20)
Test specificity	99%	99%	Beta	Diaceutics (20)
Prevalence	13.8%	2.7%	Beta	Polak et al. (21)
Standardized mortality ratio for excess mortality of BRCA mutations	1.46		Not sampled	Mai et al. (22)
Cost inputs				
Test Cost	£288.91	£288.91	Gamma	Non-comparative model
Counseling cost	£0	£0	Not sampled	Non-comparative model
Total (discounted) costs for TP (gBRCAm/Olaparib)	£58,782	£69,204	Gamma	Non-comparative model
Total (discounted) costs for FP (gBRCA wild type [gBRCAwt]/Olaparib)	£58,782	£69,204	Gamma	Non-comparative model
Total (discounted) costs for TN (gBRCAwt/SoC)	£9,548	£19,275	Gamma	Non-comparative model
Total (discounted) costs for FN (gBRCAm/SoC)	£9,512	£19,031	Gamma	Non-comparative model
Total (discounted) costs for gBRCAwt in the no testing scenario on the SoC	£9,548	£19,275	Gamma	Non-comparative model
Total (discounted) cost for gBRCAm in the no testing scenario on the SoC	£3,831	£3,831	Gamma	Non-comparative model
Health benefit inputs				
Total (discounted) LYs for TP (gBRCAm/Olaparib)	17.72	15.18	Normal	Non-comparative model
Total (discounted) LYs for FP (gBRCAwt/Olaparib)	17.04	14.43	Normal	Non-comparative model
Total (discounted) LYs for TN (gBRCAwt/SoC)	17.04	14.43	Normal	Non-comparative model
Total (discounted) LYs for FN (gBRCAm/SoC)	16.35	14.00	Normal	Non-comparative model
Total (discounted) LYs for gBRCAwt in the no testing scenario on the SoC	17.04	14.43	Normal	Non-comparative model
Total (discounted) LYs for gBRCAm in the no testing scenario on the SoC	16.35	14.00	Normal	Non-comparative model
Total (discounted) QALYs for TP (gBRCAm/Olaparib)	14.34	12.32	Normal	Non-comparative model
Total (discounted) QALYs for FP (gBRCAwt/Olaparib)	13.70	11.66	Normal	Non-comparative model
Total (discounted) QALYs for TN (gBRCAwt/SoC)	13.70	11.66	Normal	Non-comparative model
Total (discounted) QALYs for FN (gBRCAm/SoC)	13.21	11.34	Normal	Non-comparative model
Total (discounted) QALYs for gBRCAwt in the no testing scenario on the SoC	13.70	11.66	Normal	Non-comparative model
Total (discounted) QALYs for gBRCAm in the no testing scenario on the SoC	13.21	11.34	Normal	Non-comparative model


True positive (TP)=Sensitivity×Prevalence

True negative (TN)=(1−Prevalence)×Specificity

False positive (FP)=(1−Prevalence)−TN

False negative (FN)=Prevalence−TP

Conditional share formulae were used to construct conditional outcomes for the olaparib and SoC arms under the “test and treat” scenario. The total costs and health benefits for each treatment branch were calculated as follows:


$$\begin{array}{c} Olaparib\;Total\;Costs\\ =\frac{TP}{TP+FP}\times TP\;Costs+\frac{FP}{TP+FP}\times FP\;Costs+Test\;Cost \end{array}$$

$$\begin{array}{c} Olaparib\;Total\;QALY\\ =\frac{TP}{TP+FP}\times TP\;QALY+\frac{FP}{TP+FP}\times FP\;QALY \end{array}$$

$$\begin{array}{c} SoC\;Total\;Cost=\frac{TN}{TN+FN}\times TN\;Costs+\frac{FN}{TN+FN}\times FN\;Costs\\ +Test\;Cost \end{array}$$

$$\begin{array}{c} SoC\;Total\;QALY\\ =\frac{TN}{TN+FN}\times TN\;QALY+\frac{FN}{TN+FN}\times FN\;QALY \end{array}$$

Unconditional outcomes for the BRCA “test and treat” and “no testing” scenarios were then calculated:


$$\begin{array}{c} Expected\;Total\;QALY\;Test\;and\;Treat\\ \;=Olaparib\;Treatment\;QALY\times \left(TP+FP\right)\\ +SOC\;Treatment\;QALY\times \left(TN+FN\right) \end{array}$$

$$\begin{array}{c} Expected\;Total\;Costs\;Test\;and\;Treat\\ =Olaparib\;Total\;Cost\times \\ \left(TP+FP\right)+SOC\;Total\;Cost\times \left(TN+FN\right) \end{array}$$

$$\begin{array}{c} Expected\;QALY\;No\;Testing=Prevalence\\ \times FN\;QALY+\left(1-Prevalence\right)\times TN\;QALY \end{array}$$

$$\begin{array}{c} Expected\;Costs\;No\;Testing=Prevalence\\ \times FN\;Cost+\left(1-Prevalence\right)\times TN\;Cost \end{array}$$

By subtracting the cost and QALYs for “no testing” from those for “test and treat”, we obtained the incremental costs and health benefits associated with BRCA “test and treat” for the entire population of patients with HER2- high-risk eBC.

Health benefit payoffs were measured in total (discounted) LYs and QALYs. Costs were measured in total (discounted) treatment costs and the unit cost of BRCA testing. The economic value of BRCA testing plus treatment with olaparib was measured using the incremental cost per additional QALY gained (ICER/QALY).

### Sensitivity analysis

We conducted a one-way sensitivity analysis (OWSA) to assess the uncertainty surrounding the model inputs and pinpoint the key drivers of cost-effectiveness (CE) results. In a deterministic framework, each parameter was individually varied within a specified lower and upper bound with the resulting changes in the analysis documented. For test cost and gBRCAm prevalence, the bounds were set based on a 10% variability of the base case parameter. A smaller 1% variability was applied to cost-effectiveness model (CEM) payoffs, as these were derived from a model dependent on multiple inputs simultaneously. The OWSA results for the 10 most influential parameters were illustrated using a tornado diagram, which displayed the variability in ICER results, ordered by the extent of variation (with the greatest variation at the top). We also performed a probabilistic sensitivity analysis (PSA) to estimate the full parametric uncertainty surrounding the results of the cost-effectiveness analysis. The PSA was conducted through the repeated re-sampling of all major input parameters using stochastic distributions to generate a series of sampled estimates of the cost-effectiveness results under uncertainty. For most parameters, the bounds were based on the 95% confidence interval of the input parameter. The distributions used for each parameter are shown in **Table 1**. The PSA results are presented in a scatterplot of ICERs and a cost-effectiveness acceptability curve, generated by plotting the proportion of simulated ICERs in which the intervention was cost-effective (y-axis) against a variable willingness-to-pay threshold (x-axis).

## Results

### Base case

In the global base case for patients with TNBC, BRCA testing and adjuvant olaparib treatment were associated with an incremental gain in QALY of 0.15 and an incremental cost of £7,581, compared to no testing and the SoC, leading to an ICER/QALY of £49,327 (**Table 2**).

**Table 2:** Base case results.

Intervention	Total costs (£)	Total QALYs	ICER/QALY (£)
TNBC			
BRCA testing and adjuvant olaparib	£17,125	13.79	–
No testing and the SoC	£9,543	13.63	–
Incremental results	£7,581	0.15	£49,327
HER2-/HR+			
BRCA testing and adjuvant olaparib	£21,523	11.67	–
No testing and the SoC	£19,268	11.65	–
Incremental results	£2,255	0.03	£86,349

In the global base case for patients with HER2-/HR+ eBC, BRCA testing and adjuvant olaparib treatment were associated with an incremental gain in QALY of 0.03 and an incremental cost of £2,255, compared to no testing and the SoC, leading to an ICER/QALY of £86,349 (**Table 2**).

### OWSA

**Figure 2:**
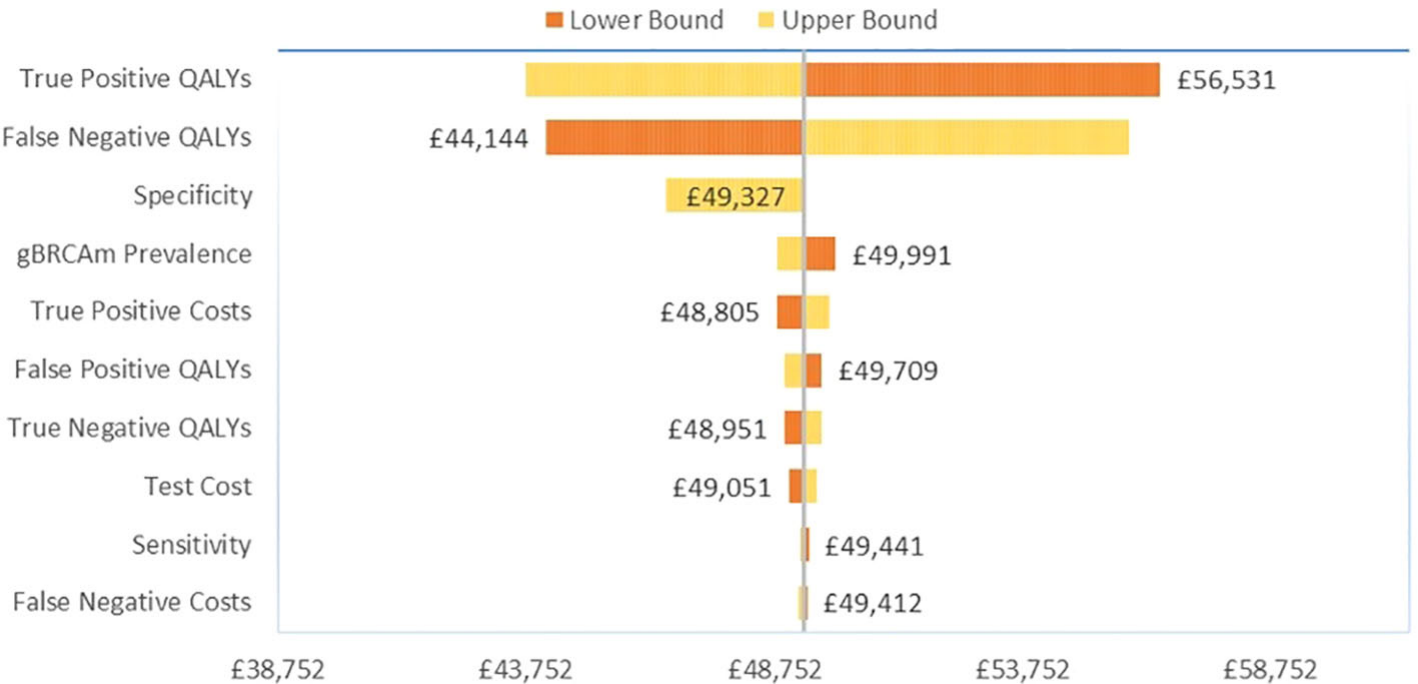
Tornado diagram for OWSA in TNBC.

Among the TNBC patients, variation in the gain in QALYs for gBRCAm patients had the greatest influence on base case results versus no testing (**Figure 2**). For true positive gBRCAm, this was the resulting gain in QALYs from receipt of olaparib therapy. For false negative gBRCAwt, this was the reduction in quality-adjusted survival under watch and wait (no testing and the SoC). Test specificity or the ability of the test to correctly identify gBRCAwt patients as gBRCAwt ranked third in terms of influence on base case results.

**Figure 3:**
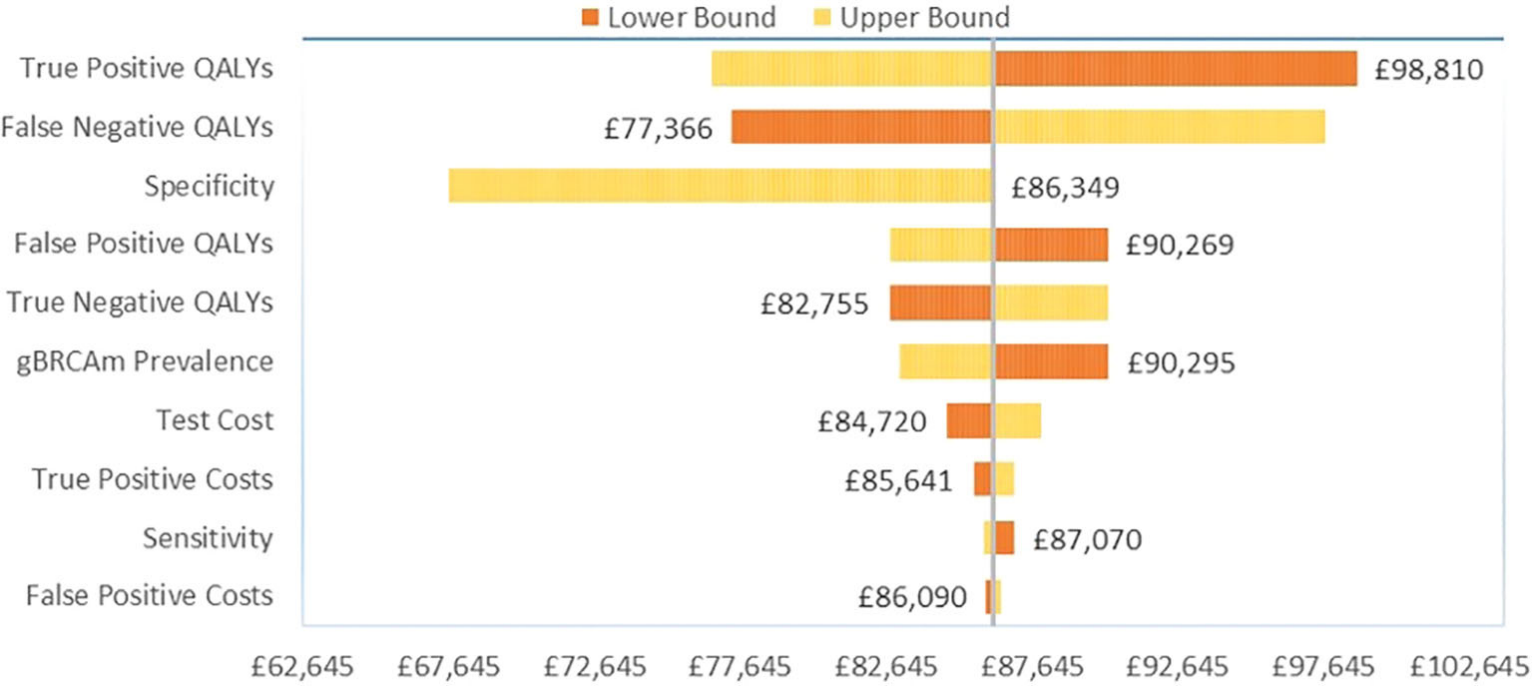
Tornado diagram for OWSA in HER2-/HR+ BC.

Among the HER2-/HR+ patients, variation in the gain in QALYs for gBRCAm patients again had the greatest influence on base case results versus no testing (**Figure 3**). For true positive gBRCAm, this was the gain in QALYs from receipt of olaparib therapy. For false negative gBRCAwt, this was the change in QALYs under watch and wait. Test specificity or the ability of the test to correctly identify gBRCAwt patients as gBRCAwt again ranked third in terms of influence on base case results.

### PSA

**Figure 4:**
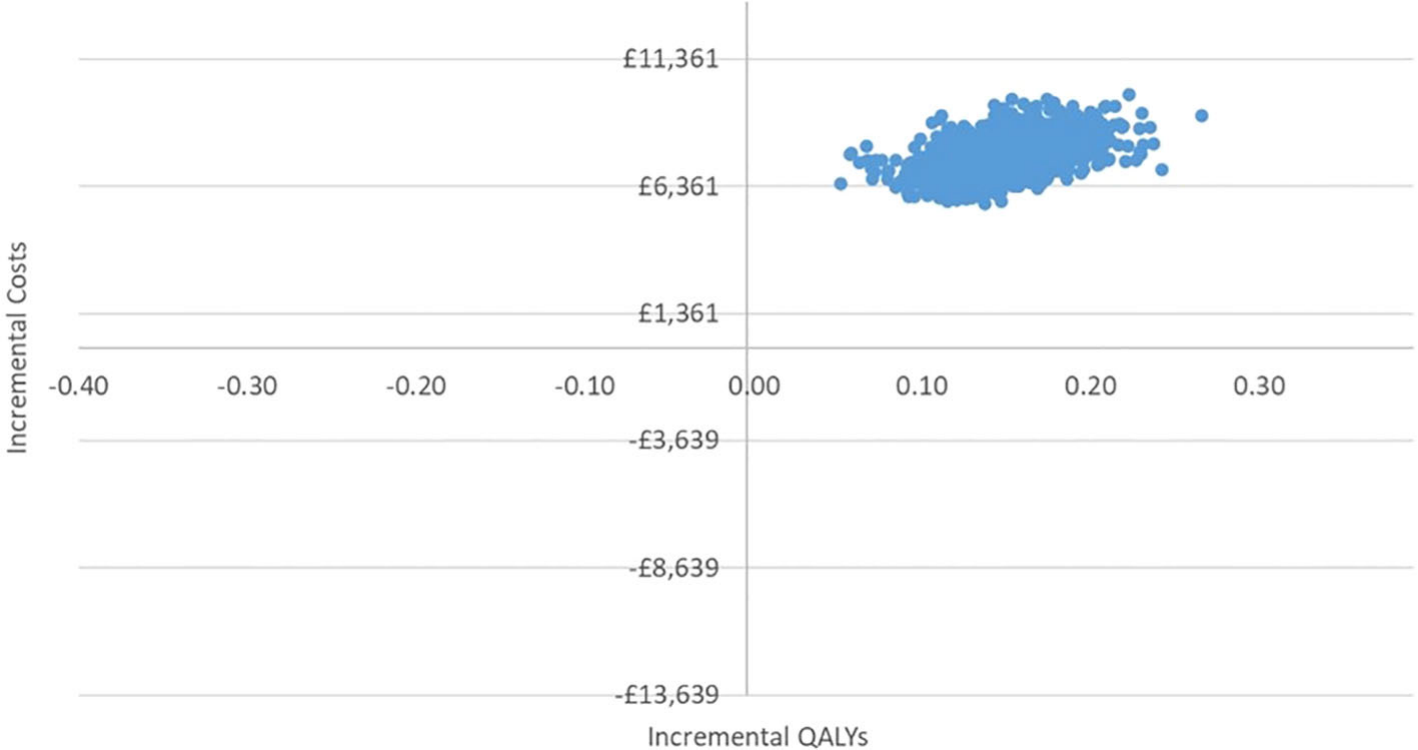
Cost-effectiveness plane for TNBC.

**Figure 5:**
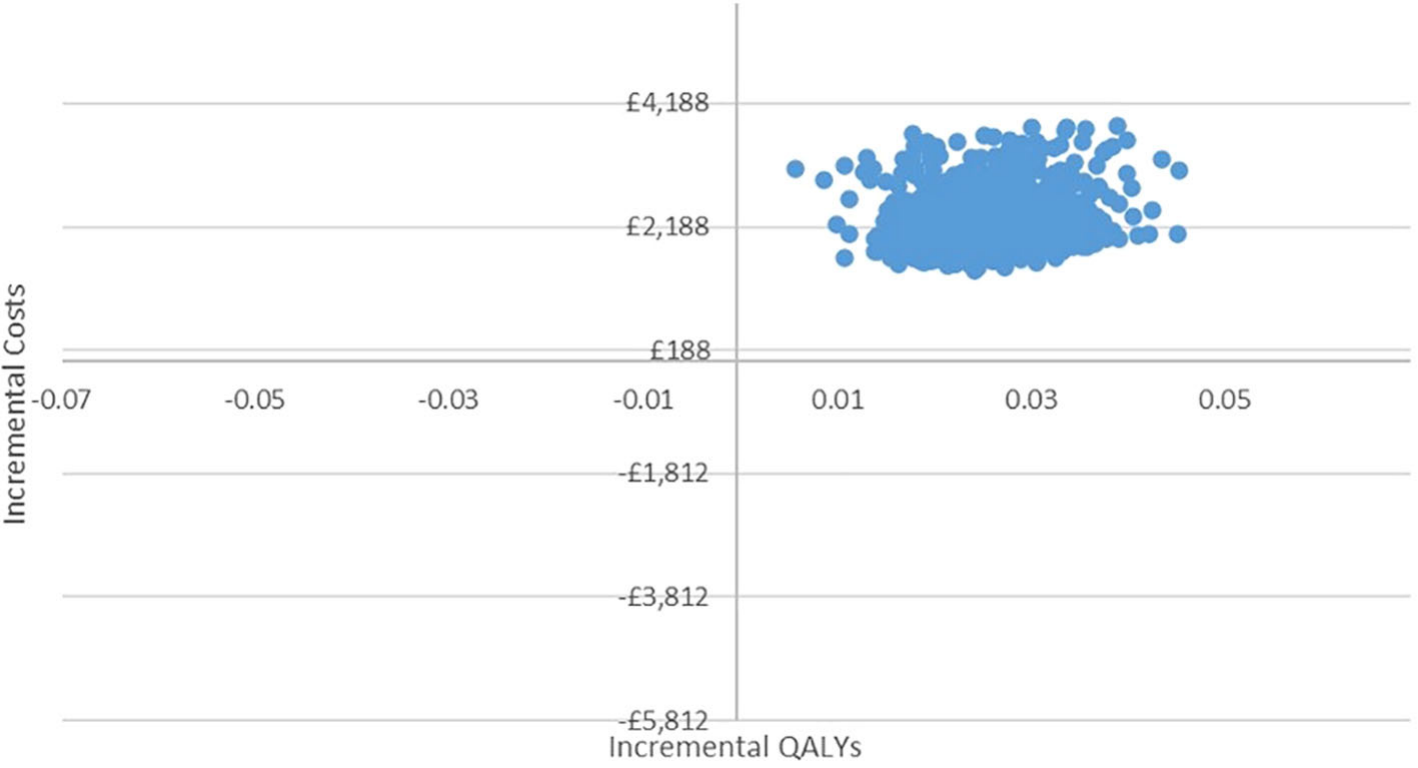
Cost-effectiveness plane for HER2-/HR+ BC.

**Figure 6:**
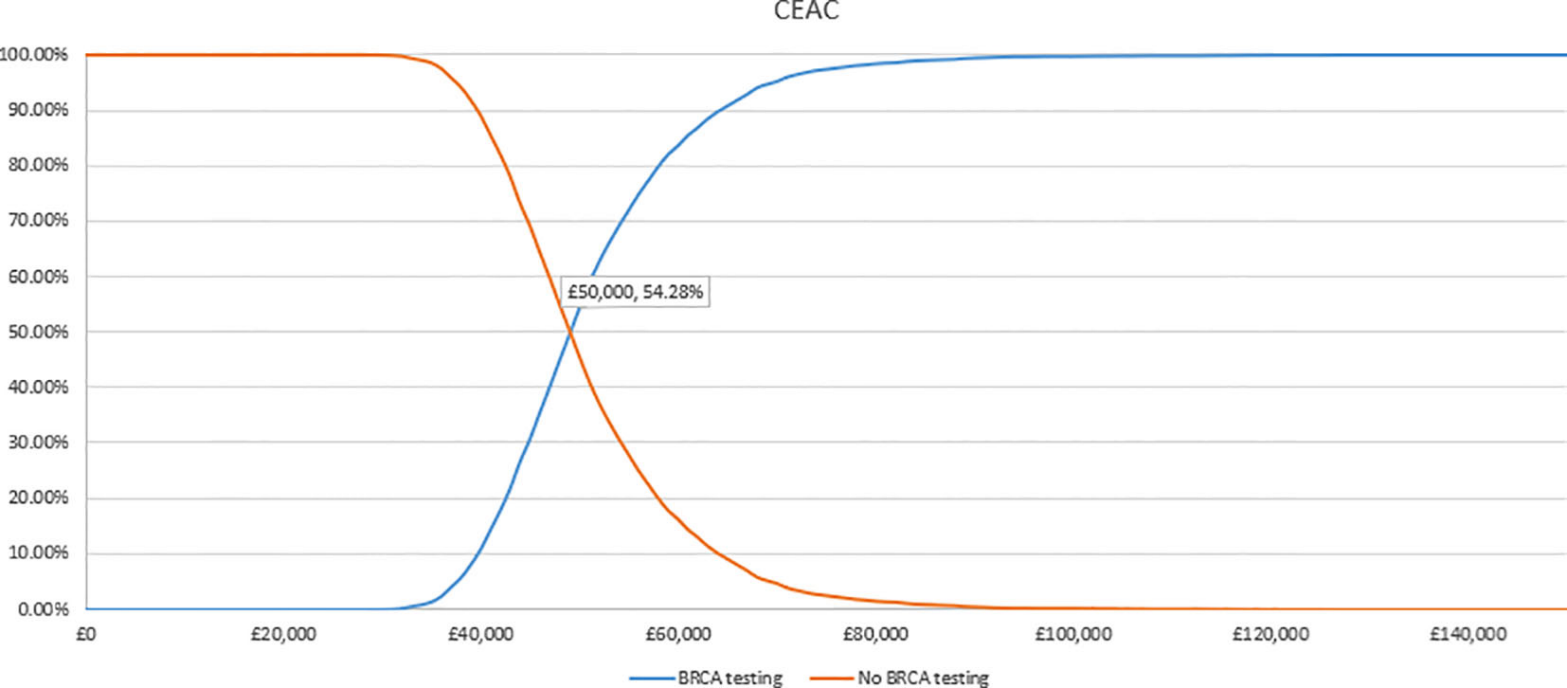
Cost-effectiveness acceptability curve for TNBC.

**Figure 7:**
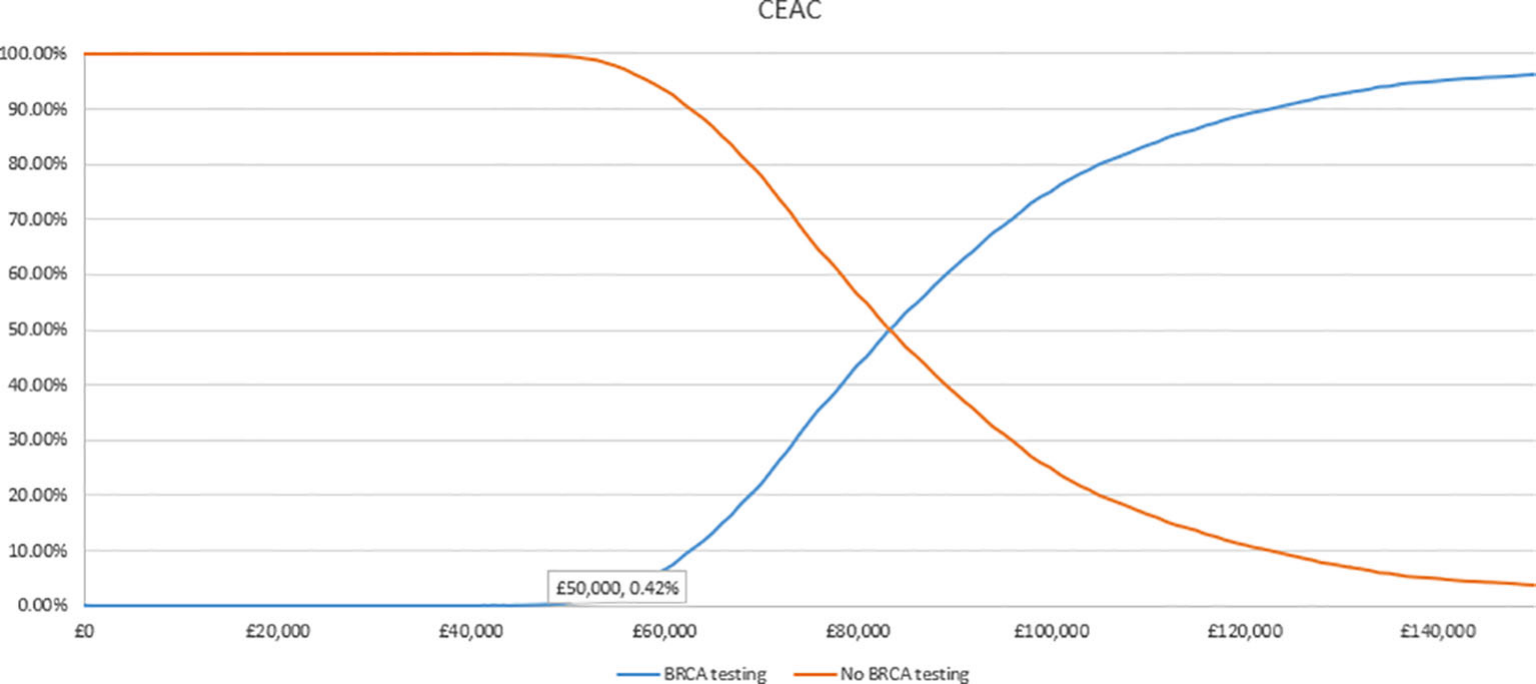
Cost-effectiveness acceptability curve for HER2-/HR+ BC.

In the PSA, we found that, using 1,000 simulations, gBRCAm testing and adjuvant olaparib were both more costly and more effective than no testing or olaparib for both (**Figures 4**, **5**). At the conventional cost-effectiveness threshold of £20,000–£30,000, however, gBRCAm testing and adjuvant were not cost-effective in either population subgroup. The innovative test and treat therapy had a 54.3% probability of being cost-effective in patients with TNBC and 0.4% in patients with HER2-/HR+ (**Figures 6**, **7**) at a higher £50,000 threshold.

## Discussion

Rapid development in diagnostic technologies in parallel with targeted therapies has enabled the transition from traditional oncology treatment to precision medicine and treatment. The genes most commonly affected in hereditary breast and ovarian cancer are the *BRCA1* and *BRCA2* genes. Approximately 3% of breast cancers (approximately 7,500 women per year) result from inherited mutations in the *BRCA1* and *BRCA2* genes (23). Olaparib is a PARP inhibitor, inhibiting poly ADP ribose polymerase (PARP), an enzyme involved in DNA repair. It acts against cancers in people with hereditary *BRCA1* or *BRCA2* mutations including breast cancer and some ovarian and prostate cancers (24).

In our base-case analysis from a UK health care system perspective, testing for gBRCAm combined with treatment with adjuvant olaparib was associated with an ICER of £49,327 per QALY gained and an ICER of £86,349 per QALY gained for patients with TNBC and HER2-/HR+, respectively, compared to no testing and treatment with the SoC for all patients. This difference lies in the relatively poor prognosis for patients with TNBC, where proper diagnosis and treatment with targeted therapy are especially beneficial. For both eBC patient subgroups, testing and olaparib improved patient outcomes and, despite its relatively high cost, the test and treat strategy was deemed to represent an acceptable use of resources. Olaparib for adjuvant treatment of patients with BRCAm HER2- eBC has been reimbursed in the UK under a commercial agreement (TA886), which makes olaparib available to the NHS at a discount and improves the cost-effectiveness of the test and treat strategy. In Canada, olaparib has been listed as the adjuvant treatment for adult patients with deleterious or suspected deleterious gBRCAm HER2-negative high-risk eBC who have been treated with neoadjuvant or adjuvant chemotherapy, with the condition that these patients must have confirmation of a germline BRCA mutation before olaparib is initiated (25).

Our analysis addressed several key considerations for the modeling of diagnostic techniques. First, the analysis focused on patients with eBC regardless of their gBRCAm status, aiming to reflect clinical practice. Second, the diagnostic costs in the model include the cost of the test and consultation costs. A further strength of the BRCA test and treat model is that it separately assessed the cost-effectiveness in the TNBC and HER2-/HR+ groups to capture any differences in long-term recurrence risk by receptor group.

Model generalizability is attained by using the outputs from the HTA cost-effectiveness model as inputs in the BRCA test and treat model. Correlations between CEM outputs were not incorporated in the testing model This restriction might potentially limit the reliability of the PSA analysis. The diagnostic model, however, gains generalizability by avoiding additional assumptions of the correlation structure. Our analysis did not include additional costs associated with staffing and material costs related to testing outside of the cost of the test itself. Accounting for the full cost of testing using a cost comparison tool can help inform local and national policymakers on the value of testing (26).

The advancement of high-throughput sequencing technologies, coupled with the rise of targeted treatments in recent years, has facilitated a shift from conventional medical practices to individualized oncology therapeutic approaches. Utilizing extensive genomic testing, patients can receive more tailored treatments that minimize adverse effects, embodying the essence of precision medicine. Precision medicine signifies a transformative shift in healthcare, necessitating the collective involvement of all key players—healthcare providers, academia, policymakers, industry, and patients—in this systemic overhaul. The goal is to forge a cohesive, non-compartmentalized precision healthcare ecosystem that serves both patients and society comprehensively. The need for practical assessment models and tools for sequencing technologies become key requirements to drive PM forward. Our analysis presented the value of combining genetic sequencing and targeted therapy for patients with BC with gBRCAm and also provided the prototype of a testing model that can be utilized to promote PM for better patient outcomes.

## Data Availability

All data used in the analysis are presented in **Table 1** of the article.
